# *OSR1* and *SIX2* drive divergent transcriptional programs in human kidney cells: implications for regeneration and tumorigenesis

**DOI:** 10.3389/fbioe.2025.1645499

**Published:** 2025-10-03

**Authors:** Naomi Pode-Shakked, Osnat Cohen-Zontag, Dorit Omer, Orit Harari-Steinberg, Einav Vax, Oren Pleniceanu, Benjamin Dekel

**Affiliations:** ^1^ Pediatric Nephrology, Dana Dwek Children’s Hospital, Tel Aviv Medical Center, Tel Aviv, Israel; ^2^ Laboratory for Human Kidney Development Research under the Center of Regeneration and Longevity (CORAL), Tel Aviv Medical Center, Tel Aviv, Israel; ^3^ Sagol Center for Regenerative Medicine, Tel Aviv University, Tel Aviv, Israel; ^4^ Gray Faculty of Medical & Health Sciences, Tel Aviv University, Tel Aviv, Israel; ^5^ Pediatric Stem Cell Research Institute and Division of Pediatric Nephrology, Edmond and Lily Safra Children’s Hospital, Sheba Medical Center, Tel Hashomer, Israel; ^6^ Tissue Engineering Research Laboratory, Sheba Medical Center, Ramat-Gan, Israel; ^7^ Kidney Research Lab, The Institute of Nephrology and Hypertension, Sheba Medical Center, Tel Hashomer, Israel

**Keywords:** nephron progenitor cell, kidney development, reprogramming, Wilms’ tumor, human adult kidney, kidney regeneration

## Abstract

**Background:**

The nephron progenitor cells generate approximately one million nephrons during human nephrogenesis. At 34-36 weeks of human genstation, silencing of the key kidney progenitor genes results in depletion of this progenitor pool, limiting the regeneration capacity of the mature kidney. Concurrently, the increasing incidence of end-stage kidney disease underscores the urgent need for innovative regenerative strategies.

**Methods:**

We employed lentiviral vectors to ectopically induce two key kidney progenitor genes *OSR1* and *SIX2* individually or together in primary human adult kidney (hAK) cells. We then analyzed the cellular and molecular consequences through morphological assessments, functional assays, *in vivo* transplantation studies, and comprehensive transcriptional profiling.

**Results:**

*OSR1* and *SIX2* induced distinct reprogramming processes with differential functional outcomes; *SIX2* overexpression was found to maintain epithelial morphology while significantly enhancing proliferation and clonogenic efficiency. Transcriptionally, *SIX2* established epithelialization and cell-cycle networks by downregulating proximal tubule markers while upregulating distal nephron markers and proliferation genes. *In vivo*, *SIX2*-expressing cells formed organized tubular structures with a distinct luminal architecture in a proof-of-concept model. In contrast, *OSR1* overexpression was found to induce morphological changes and activate developmental morphogenetic pathways, including epithelial tube morphogenesis and canonical *Wnt* signaling; however, it did not enhance proliferation and showed minimal tubulogenic capacity *in vivo*. Unexpectedly, *OSR1* overexpression led to malignant transformation in one clone and exhibited Wilms’-tumor-like features, including expression of kidney developmental markers (i.e., SIX2, NCAM1, and WT1) and blastemal phenotype.

**Conclusion:**

Our findings suggest that *SIX2* overexpression in primary hAK cells functionally confers enhanced self-renewal and tubulogenic capacity while transcriptionally inducing a proximal-to-distal tubular cell diversion with maintained proliferative programs. In contrast, *OSR1* activates the broader developmental morphogenetic networks but poses potential oncogenic risks. The malignant transformation observed with *OSR1* overexpression provides insights into the potential cellular origins of Wilms’ tumor and raises important safety considerations for regenerative medicine approaches involving developmental gene induction in adult kidney cells.

## Introduction

End-stage kidney disease presents a major global health challenge and has limited therapeutic options, such as dialysis and transplantation (Humphreys, 2018; Freedberg et al., 2024). The human adult kidney (hAK) possesses a limited capacity for regeneration, primarily owing to the cessation of nephrogenesis before birth and the subsequent silencing of key developmental genes, which limit the ability to form new nephrons (Little and McMahon, 2012; Huang et al., 2024). This limitation motivates the development of regenerative medicine approaches aimed at restoring kidney function, with a focus on recapitulating the developmental processes. Harnessing controlled regeneration also offers a promising avenue for treating acute kidney injury (AKI), with the aim of minimizing aberrant repair and progression to chronic kidney disease (CKD) (Humphreys, 2018; Bahrami et al., 2024; Hendry et al., 2013).

During development of the kidney, the nephron progenitor cells (NPCs) characterized by the expressions of transcription factors like OSR1, SIX2, and PAX2 enable the formation of functional nephrons (Little and McMahon, 2012; Huang et al., 2024; Hendry et al., 2013; Kobayashi et al., 2008; Brodbeck and Englert, 2004; Andrianova et al., 2019). Among these factors, OSR1 and SIX2 have highly specialized roles and are pivotal regulators of nephron patterning. SIX2 is a critical factor that maintains the self-renewal and stemness of the NPC population throughout kidney development, preserving this progenitor pool during nephrogenesis. Genetic ablation of SIX2 results in premature depletion of the NPCs and subsequent kidney hypoplasia, underscoring its essential role in kidney formation. However, OSR1 is a key determinant of early–intermediate mesoderm patterning and subsequent nephron progenitor specification. OSR1 interacts synergistically with SIX2 to reinforce the nephron progenitor state by regulating the balance between progenitor maintenance and differentiation (Xu et al., 2014). The coordinated actions of these transcription factors create a precise regulatory network that controls nephron formation and patterning during embryonic development (Xu et al., 2014; O'Brien et al., 2018; O'Brien et al., 2016).

Given the absence of resident NPCs in the adult kidney, effective regeneration strategies often necessitate an initial dedifferentiation step in mature kidney cells to regain a progenitor-like state before inducing appropriate differentiation into functional nephron components (Andrianova et al., 2019; Romagnani et al., 2013; Little and Kairath, 2016; Oxburgh et al., 2017). Improving the self-renewal capacity of adult kidney cells is a critical step toward effective kidney regeneration. While the genetic network regulating nephrogenesis involves factors such as PAX/Eya/Six (Brodbeck and Englert, 2004), re-expression of the pluripotency genes offers a potential strategy to restore components that are lost or limited in adult kidney tissues, such as self-renewal. For instance, *OCT4* is an established pluripotency gene and one of the Yamanaka factors used for reprogramming somatic cells into induced pluripotent stem cells (iPSCs); it has been shown to induce the formation of dedifferentiated kidney progenitors from adult kidney cells that retain the potential to further differentiate into kidney lineages, providing a potential strategy for renal regeneration (Brodbeck and Englert, 2004; Mikkelsen et al., 2008; Omer et al., 2023; Montserrat et al., 2016; Morizane et al., 2017; Tanigawa and Nishinakamura, 2016). Although re-expression of the developmental genes in mature kidney cells holds promise for kidney regeneration, previous approaches focusing on proximal tubule cells as the source have had limited success in achieving sustained reprogramming and complete differentiation (Hendry et al., 2013). Furthermore, a crucial concern remains regarding the potential for malignant transformation, given the link between aberrant developmental processes and tumorigenesis (Hendry et al., 2013). Wilms’ tumor (WT), which is the most common pediatric renal malignancy, arises from fetal kidney stem cells that have undergone differentiation arrest (Pode-Shakked and Dekel, 2011). Aberrant developmental pathways can lead to uncontrolled proliferation and tumor formation (Aiden et al., 2010; Fukuzawa et al., 2017; Hohenstein et al., 2015; Shankar et al., 2022).

In the present study, we investigated the effects of ectopic expressions of *OSR1* and *SIX2*, both individually and in combination, in hAK cells. By reintroducing these master regulators of nephron development, we aimed to assess their capacities to induce NPC-like states and restore the nephron-forming potential of mature kidney cells. To evaluate the functional consequences of *OSR1* and *SIX2* re-expressions, we conducted comprehensive analyses of the cellular morphology, proliferation capacities, gene expression profiles, and *in vivo* tubulogenic potential. Concomitantly, we found that ectopic expression of *OSR1* resulted in malignant transformation, generating Wilms’-tumor-like tissue in one out of 100 transduced clones following transplantation into immunodeficient mice. By characterizing these processes, we aim to contribute toward better understanding of the functional effects of these two pivotal nephron patterning genes and their potential applications in regenerative medicine approaches for kidney diseases.

## Materials and methods

### Primary hAK cell cultures

Normal hAK samples were retrieved from the borders of renal cell carcinoma tumors from patients undergoing partial or total nephrectomy at Sheba Medical Center. This procedure was performed after approval by the local ethical committee and after obtaining signed informed consent from the patients (smc-5574-18). The samples were minced in Hanks’ balanced salt solution, soaked in collagenase for 2 h, and cultured in a growth medium consisting of Iscove’s modified Dulbecco’s medium supplemented with 10% fetal bovine serum (FBS), 1% L-glutamine, 1% penicillin–streptomycin, and the following growth factors: 50 ng/mL of bFGF, 50 ng/mL of EGF, and 5 ng/mL of SCF (R&D Systems, Minneapolis, MN, United States) at 37 °C in a humidified atmosphere containing 5% CO_2_. The cells were passaged using trypsin–EDTA (Gibco) upon reaching 70%–80% confluency and used between passages 2 and 7 for all experiments to ensure consistency in cellular phenotype while avoiding senescence-associated changes.

### Lentiviral vector construction and transduction

Lentiviral vectors encoding human *OSR1* and *SIX2* cDNA under the control of the cytomegalovirus promoter were purchased from Vector Builder. The *OSR1* construct included an mCherry fluorescent reporter for visual confirmation of expression, whereas the *SIX2* construct contained only the gene of interest. A lentiviral vector encoding mCherry alone was used as a control to account for any effects of viral transduction or fluorescent protein expression. For transduction, the hAK cells were seeded at a density of 5 × 10^4^ cells per well in 6-well plates and allowed to adhere for 24 h. The cells were then transduced with lentiviral particles at a multiplicity of infection (MOI) of 5 in the presence of 8 μg/mL polybrene (Sigma-Aldrich) for enhanced transduction efficiency. After 24 h of incubation with the viral particles, the medium was replaced with fresh renal epithelial cell growth medium containing 10% FBS to remove any residual viral particles and polybrene. The transduced cells were selected with puromycin (2 μg/mL) for 7 d to establish stable cell lines expressing the genes of interest. To generate double-transduced cells, *OSR1*-expressing cells were additionally transduced with *SIX2*-encoding virus and selected with appropriate antibiotics (puromycin and neomycin) to ensure the expression of both factors. The resulting cell lines were designated as OSR1-hKEpCs, SIX2-hKEpCs, OSR1+SIX2-hKEpCs, and mCherry-hKEpCs (Supplementary Table S1). Successful transduction was confirmed through quantitative polymerase chain reaction (PCR), Western blotting, and fluorescence microscopy for the mCherry reporter.

### Quantitative real-time PCR (qRT-PCR)

The total RNA was extracted from the hAK cells using the RNeasy Mini Kit (Qiagen) according to the manufacturer's instructions. The RNA concentration and purity were then assessed using a NanoDrop spectrophotometer (Thermo Fisher Scientific), and samples having 260/280 ratios between 1.8 and 2.0 were considered suitable for further analyses. Next, cDNA was synthesized using the iScript cDNA Synthesis Kit (Bio-Rad) with 1 μg of RNA as the template. Real-time PCR was performed using the CFX96 RT-PCR Detection System (Bio-Rad) with SYBR Green PCR Master Mix (Bio-Rad). The thermal cycling conditions consisted of initial denaturation at 95 °C for 3 min, followed by 40 cycles of denaturation at 95 °C for 15 s each, annealing at 60 °C for 30 s, and extension at 72 °C for 30 s. A melting curve analysis was performed to confirm the specificity of the amplified products. Primers for all the target genes were designed using Primer-BLAST (NCBI) and validated for specificity and efficiency prior to use. The gene expressions were normalized to glyceraldehyde-3-phosphate dehydrogenase (GAPDH) using the 2^-ΔΔCt^ method. Three independent experiments were performed with triplicate samples for each condition to ensure reproducibility.

### Cell proliferation and clonogenic assays

For the proliferation assays, the cells were seeded at a density of 2 × 10^4^ cells per well in 12-well plates with complete growth medium. The cells were then counted at 24-h intervals for 5 d using a hemocytometer after trypsinization and trypan blue dye exclusion to assess viability. The growth rate was calculated as the number of cell divisions per day normalized to the control cells to determine the relative proliferation rate. For the clonogenic assays, the cells were seeded at ultralow densities (0.3, 1, and 10 cells per well) in 96-well plates via limiting dilution. After 14 d of culture with medium changes every 3 d, the colonies were fixed with 4% paraformaldehyde for 15 min at room temperature, stained with 0.5% crystal violet for 30 min, and washed thoroughly with phosphate-buffered saline (PBS). Colonies containing more than 50 cells were then counted under a light microscope, and the clonal efficiency was calculated as the percentage of wells containing colonies relative to the total number of wells seeded. For each cell line, at least 96 wells were analyzed for each density condition across three independent experiments.

### *In vivo* transplantation for tubulogenesis assays

All animal procedures employed in this study were approved by the Institutional Animal Care and Use Committee (IACUC-15-2018) at Sheba Medical Center and performed in accordance with the institutional guidelines. For subcutaneous transplantation, engineered human kidney epithelial cells (hKEpCs) were harvested at passages 3–6 using trypsin–EDTA (0.25%, Gibco), washed twice with PBS, and resuspended in growth-factor-reduced Matrigel (BD Biosciences) to provide a supportive extracellular matrix environment. Two injection protocols were then employed: 1.5 × 10^6^ cells in 200 μL of Matrigel for the AK83 cell line; 2 × 10^6^ cells in 50 μL of Matrigel mixed with 50 μL of PBS for the AK87 cell line. The cell suspensions were injected subcutaneously into the dorsal flank of 6–8 week old male NOD-SCID mice (n = four to six per group) under isoflurane anesthesia (2%–3% in oxygen). The injected cell masses were harvested either immediately (T0) or after 14 d (T14) for analyses. The animals were maintained under specific pathogen-free conditions at the animal facility of Sheba Medical Center with *ad libitum* access to food and water; further, they were monitored daily for signs of discomfort or health issues, and their body weights were recorded weekly.

### Immunohistochemical analysis

The tissue samples were collected at 14 d and 28 d post-injection, fixed in 4% paraformaldehyde overnight at 4 °C, processed through graded ethanol, embedded in paraffin, and sectioned with a thickness of 5 μm. For the immunofluorescence analysis, the sections were deparaffinized, subjected to antigen retrieval in citrate buffer (pH 6.0) for 20 min at 95 °C, and blocked with 5% normal goat serum for 1 h at room temperature to reduce non-specific binding. The sections were next incubated overnight at 4 °C with the following primary antibodies: anti-human leukocyte antigen (HLA; 1:100, Abcam), anti-CD13 (1:200, Abcam), anti-epithelial membrane antigen (EMA; 1:100, Dako), anti-multifunctional/mesenephric nephric marker (MNF; 1:100, Sigma), anti-vimentin (1:200, Abcam), anti-pancytokeratin (1:200, Invitrogen), anti-E-cadherin (1:200, BD Biosciences), anti-SIX2 (1:200, Proteintech), and anti-Ki67 (1:100, Cell Signaling). After washing with PBS, the sections were incubated with appropriate Alexa-Fluor-conjugated secondary antibodies (1:500, Invitrogen) for 1 h at room temperature, and the nuclei were counterstained with DAPI (Sigma). Lastly, the slides were mounted with ProLong Gold Antifade (Invitrogen) and imaged using a Zeiss LSM 700 confocal microscope with ×20 and ×40 objectives. For the immunohistochemical analyses, the sections were processed with the Ventana automated immunostainer using DAB as the chromogen, followed by hematoxylin counterstaining. Sections from the human fetal kidney (14–16 weeks) and adult kidney were used as the positive controls for marker expression patterns, whereas primary antibody omission served as the negative control. Images were then acquired using a Zeiss LSM 700 confocal microscope or an Olympus BX51 light microscope equipped with a digital camera.

### RNA sequencing and bioinformatic analysis

The total RNA was extracted from the engineered hKEpC lines (SIX2-hKEpCs, OSR1-hKEpCs, OSR1+SIX2-hKEpCs, and naïve control hKEpCs) at passage 4–6 using the RNeasy Mini Kit (Qiagen) with on-column DNase digestion (RNase-Free DNase Set, Qiagen) to eliminate genomic DNA contamination. Three independent biological replicates were prepared for each cell line to ensure statistical robustness. Then, the RNA concentrations were measured using a NanoDrop 2000 spectrophotometer (Thermo Fisher Scientific), where 260/280 ratios between 1.8 and 2.0 were considered acceptable. RNA integrity was assessed using an Agilent 2100 Bioanalyzer with the RNA 6000 Nano Kit (Agilent Technologies), and only samples with RNA integrity number (RIN) values exceeding 8.0 were selected for library preparation and sequencing.

The RNA sequencing libraries were prepared from 1 μg of total RNA using the TruSeq Stranded mRNA Library Prep Kit (Illumina) according to manufacturer protocols. Briefly, the mRNA was isolated using poly-A selection before being fragmented and reverse-transcribed to generate first-strand cDNA using random hexamer primers. Then, the second-strand cDNA synthesis was performed using dUTP to maintain strand specificity. Following end repair, A-tailing, and adapter ligation, the libraries were amplified by PCR for 12–15 cycles. The library quality and concentration were assessed using the Agilent 2100 Bioanalyzer with the DNA 1000 Kit and quantified by qPCR using the KAPA Library Quantification Kit (KAPA Biosystems). The libraries were sequenced on an Illumina NovaSeq 6000 platform using paired-end 150-bp reads with a target depth of >30 million read pairs per sample to ensure adequate coverage for differential expression analysis. The raw sequencing reads (FASTQ files) were assessed using FastQC (v0.11.9) to evaluate the read quality metrics, adapter contamination, and level of sequence duplication. Quality filtering and adapter trimming were then performed using Trimmomatic (v0.39) with the following parameters: ILLUMINACLIP:TruSeq3-PE.fa:2:30:10, LEADING:3, TRAILING:3, SLIDINGWINDOW:4:15, and MINLEN:36. The high-quality trimmed reads were aligned to the human reference genome (GRCh38/hg38) using STAR aligner (v2.7.3a) with the default parameters optimized for mammalian genomes. The alignment quality was assessed using samtools (v1.10) and RSeQC (v3.0.1) to evaluate the mapping rates, read distributions, and potential biases.

Next, gene expression quantification was performed using featureCounts (v2.0.1) from the Subread package by counting reads mapping to protein-coding genes annotated in GENCODE v32; the raw count matrices were imported into R (v4.0.3) for downstream analyses. The differential gene expression analysis was conducted using DESeq2 (v1.30.0) with the default normalization and dispersion estimation procedures. Genes with low expressions (fewer than 30 counts across all samples) were filtered prior to the analyses. The differential expressions were assessed using the Wald test, with genes showing adjusted p-value <0.05 (Benjamini–Hochberg correction) and absolute log_2_(fold change) >1 being considered as significantly differentially expressed.

To benchmark the overexpression levels against physiological ranges, we compared the *SIX2* and *OSR1* expressions in our engineered cell lines with published RNA sequencing data from human fetal kidney tissues. The expression data were obtained from O'Brien et al. (2016), which provided the transcriptomic profiles of 17-week-old human fetal kidney cortex, including whole tissue, cortex-enriched fractions, and purified ITGA8+ NPCs isolated by FACS. The transcripts per million (TPM) values for *SIX2* and *OSR1* were extracted from published datasets and compared with our engineered cell lines to evaluate the biological relevance of the overexpression levels relative to developmental kidney cell populations. Principal component analysis (PCA) was performed using the plotPCA function in DESeq2 with variance-stabilizing transformation (VST) to visualize the global transcriptional relationships between the samples. Hierarchical clustering analysis was then conducted using the pheatmap package (v1.0.12), with Euclidean distance and complete linkage clustering of the top-1,000 most variable genes. Pearson correlation coefficients were calculated between the log_2_-normalized SIX2 or OSR1 counts and expressions of all genes (VST scale) across the samples (control and overexpressing). For each gene, the p-values were computed by permutation and adjusted for multiple testing. Genes with strong positive (r > 0.7) or negative (r < −0.7) correlations were visualized in color-coded bar plots (red: positive; blue: negative; gray: weak or non-significant). To identify genes whose expressions varied with *SIX2* or *OSR1* dosage across all samples, we fit a linear model for each gene using the limma package (lmFit, eBayes) by modeling the gene expression as a function of the log_2_ expression of SIX2 or OSR1 as well as applying empirical Bayes moderation and array weights to handle the sample outliers; genes with significant associations (adjusted p-value <0.05, |log_2_FC| > 0.1) were then selected for the downstream analyses. Gene ontology (GO) biological process (BP) enrichment was performed for the upregulated (positive slope) and downregulated (negative slope) genes using clusterProfiler enrichGO; bar plots were used to summarize the most enriched GO-BP terms (blue: upregulated; red: downregulated). The TPM values were calculated and visualized as mean ± standard error of the mean (SEM) bar plots for selected genes across different conditions. The data were visualized using ggplot2 (v3.3.2) for the PCA plots and volcano plots, pheatmap for heatmaps, and custom R scripts for the correlation plots and pathway visualizations. All bioinformatics analyses were performed using R/Bioconductor packages, along with documentation of the reproducible code for transparency and replication.

### Karyotyping

The OSR1-hKEpCs were cultured to 70%–80% confluence and treated with colcemid (0.1 μg/mL) for 2 h to arrest the cells in metaphase; the cells were then trypsinized, treated with a hypotonic solution (0.075 M of KCl) for 20 min at 37 °C, and fixed with freshly prepared methanol/acetic acid (3:1) through three sequential changes of the fixative. The fixed cell suspensions were placed dropwise onto clean glass slides and air-dried. Then, the chromosomes were stained with Giemsa (4% in phosphate buffer, pH 6.8) for 10 min and analyzed under a light microscope with a ×100 oil-immersion objective. At least 20 metaphase cells were analyzed for each sample to identify the numerical and structural chromosomal abnormalities. The karyotyping was performed in accordance with the guidelines of the International System for Human Cytogenetic Nomenclature.

### Insertion site analysis

Genomic DNA was extracted from the OSR1-hKEpC-E09 cells using the DNeasy Blood & Tissue Kit (Qiagen). Then, lentiviral integration sites were identified using custom biotinylated probes targeting the LTR regions, followed by targeted sequence capture and next-generation sequencing on an Illumina platform (Miyazato et al., 2016; Ustek et al., 2012). The sequencing reads were processed using a bioinformatics pipeline for integration site analysis, including quality filtering, genome alignment (GRCh38), and junction identification (Park et al., 2015). The insertion site was mapped to an intronic region of HECTD4 on chromosome 12, with no integration events detected in the known oncogenes or tumor suppressor genes, indicating that the malignant transformations were not due to insertional mutagenesis.

### Statistical analysis

The data are presented as mean ± SEM unless indicated otherwise in the figure legends. For the proliferation assays, experiments were performed using cells from three independent primary kidney cell isolations (n = 3 biological replicates), where each experiment was conducted in three technical replicates and repeated three times. For the clonogenic assays, three independent primary cell sources were used (n = 3 biological replicates), where each density condition analyzed in at least 96 wells per experiment and repeated across three independent experiments. For the *in vivo* transplantation studies, three different primary cell sources were used (hAK83, hAK86, and hAK87; n = 3 biological replicates), where each cell line transplanted into 4–6 mice per group. Statistical significance was determined using appropriate tests based on the data distribution and experimental design. For two-group comparisons, unpaired two-tailed Student’s t-test was used after confirming normal distribution using the Shapiro–Wilk test. For multiple groups, we performed one-way ANOVA followed by Tukey’s *post hoc* test for multiple comparisons. For the RNA sequencing data, differential expressions were assessed using DESeq2 with Benjamini–Hochberg correction for multiple testing, where adjusted p-value <0.05 and |log_2_FC| >1 were considered to be statistically significant. The exact p-values are reported in the figures where space permits, given the following significance thresholds: *p < 0.05, **p < 0.01, and ***p < 0.001. All statistical analyses were performed using GraphPad Prism 10 (GraphPad Software, San Diego, CA, United States).

The sample size was determined using the resource equation method (Festing and Altman, 2002) that is recommended when precise estimates of the effect size and variance are not available, as in exploratory mechanistic *in vivo* tubulogenesis studies. In our design, three experimental groups (naïve, *SIX2*-overexpressing, and *OSR1*-overexpressing cells) were each generated from three independent human donors (biological replicates) and transplanted into 4–6 recipient mice per group. For the resource equation *E* = *N*−*G*, where *N* is the total number of experimental units (mice) and *G* is the number of groups, our experimental range (total *N* = 12–18; *G* = 3) yielded *E* = 9–15, which is within the recommended range of 10–20 for adequately powered exploratory studies. This approach balances the need for statistical validity with ethical considerations of animal use, in line with ARRIVE guidelines. The sample sizes were determined through preliminary experiments and a power analysis to ensure adequate statistical power (>0.8) while minimizing animal usage in accordance with the ethical guidelines. All experiments included appropriate controls and were performed by investigators blinded to the experimental conditions where feasible.

## Results

### Overexpression of renal progenitor genes OSR1 and SIX2 induces distinct morphological and transcriptional changes in human kidney epithelial cells

**FIGURE 1:**
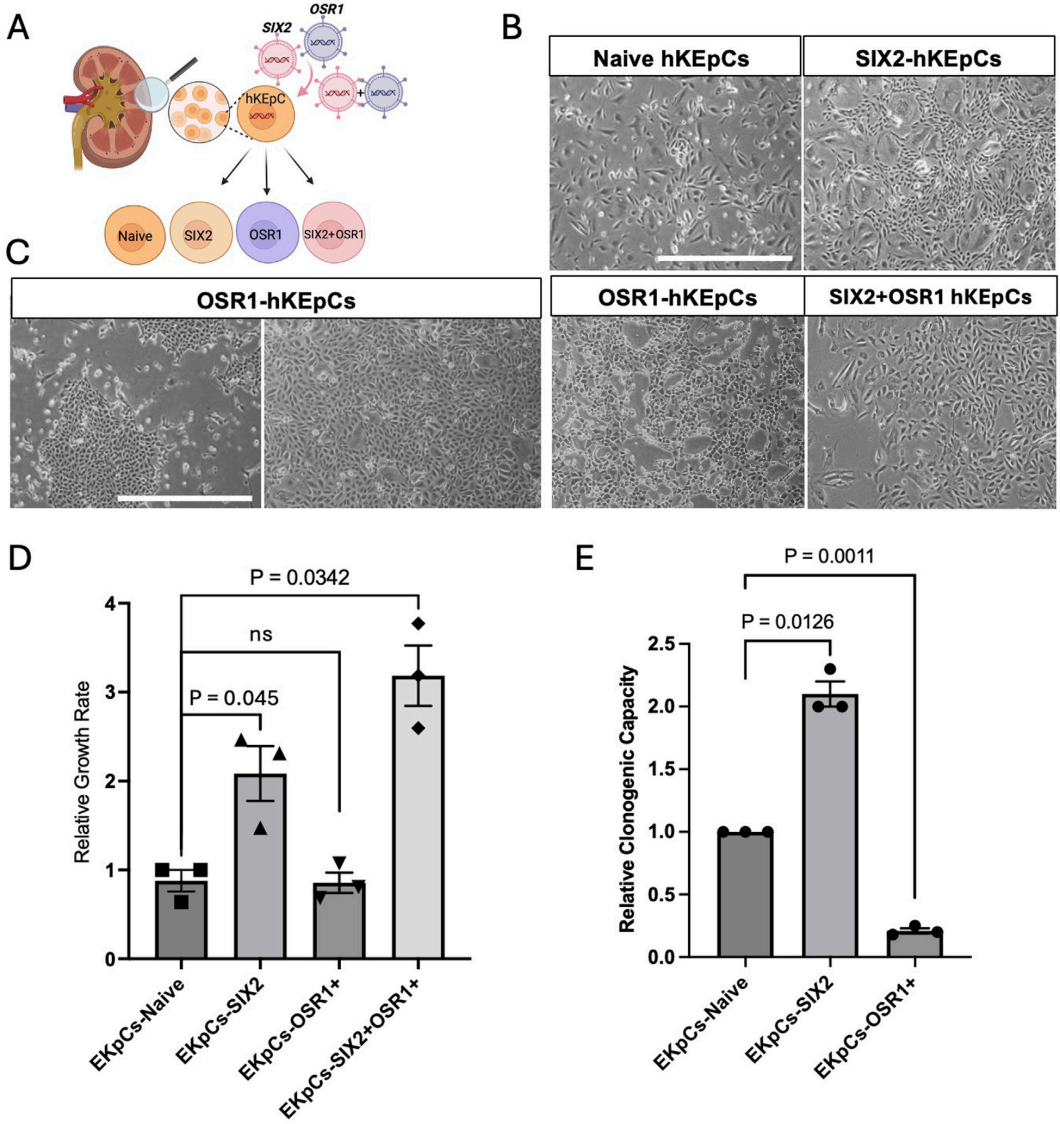
Characterization of renal-progenitor-gene-expressing human kidney epithelial cells (hKEpCs). **(A)** Schematic representation of the experimental approach. Adult hKEpCs were isolated and transduced with lentiviral vectors to express *SIX2*, *OSR1*, or both transcription factors as distinct engineered cell lines. **(B,C)** Representative phase-contrast microscopy images showing the distinct morphological characteristics of transduced hKEpCs. Naïve hKEpCs and SIX2-hKEpCs maintain a typical epithelial morphology, while OSR1-hKEpCs develop a dense hyperchromatic morphology with colony-like structures at the early passages but adopt a spindle-shaped morphology at higher passages. SIX2+OSR1 hKEpCs show an intermediate morphology. Representative images of n = 4 different primary cell sources, where each experiment was repeated at least three times. **(D)** Relative growth rates of the engineered cell lines defined as the number of cell divisions per day. The SIX2-hKEpCs and SIX2+OSR1-hKEpCs demonstrate significantly enhanced proliferations compared to the control cells. The results are presented as mean ± standard error of the mean (SEM) of n = 4 biological replicates. The *p*-values are indicated in the images. **(E)** Clonogenic capacities of the engineered cell lines measured as the ability to form colonies from single cells. The SIX2-hKEpCs show a 2.07-fold increase in colony formation compared to control cells, while the OSR1-hKEpCs demonstrate reduced clonogenic capacity. The results are presented as mean ± SEM of n = 3 biological replicates. All experiments were repeated three times, and the *p*-values are indicated in the respective images.

To investigate the differential effects of renal developmental gene induction on hAK cells, we established primary hAK cell lines overexpressing *OSR1* and *SIX2* individually and in combination (*OSR1*+*SIX2*) using lentiviral vectors (Supplementary Figure S1A). The control groups included naïve hKEpCs as well as hKEpCs expressing mCherry. Successful transduction was confirmed by qRT-PCR, which showed significant overexpressions of *SIX2* (approximately 50,000-fold) and *OSR1* (approximately 3,000-fold) compared to the control cells (Supplementary Figure S1A). *SIX2* overexpression was validated at the protein level by Western blotting, whereas *OSR1* expression was visualized through the mCherry reporter (Supplementary Figure S1B,C). The transcription factors showed appropriate nuclear localizations, confirming their functional expressions. The resultant cell lines displayed distinct morphological characteristics; the control cells (naïve hKEpCs and mCherry-hKEpCs) exhibited typical epithelial morphology characteristic of primary cultured kidney cells (Figure 1B; Supplementary Figure S1D), whereas the SIX2-hKEpCs maintained their epithelial appearance with minimal morphological alterations and showed only a slightly more compact hyperchromatic phenotype (Figure 1B). In contrast, OSR1 induction triggered significant morphological changes, with the cells developing a dense hyperchromatic morphology that was distinctly different from the surrounding cells (Figure 1C). After several weeks in culture, the OSR1-hKEpCs formed well-defined colonies with small and cuboidal cells that aggregated in colony-like structures. Notably, at higher passages, these cells adopted a spindle-shaped morphology characteristic of senescent hKEpCs, suggesting the transient nature of morphological reprogramming. The OSR1+SIX2-hKEpCs exhibited an intermediate morphology with features characteristic of overexpression of both of the primary individual genes (Figure 1B). These morphological alterations, particularly in the OSR1-hKEpCs, are consistent with activation of partial epithelial-to-mesenchymal transition (EMT), which is associated with developmental reprogramming and is observed during the early stages of nephrogenesis when mesenchymal condensation occurs prior to epithelialization.

### Differential effects on the proliferation and clonogenic capacities

The key phenotypic differences among the NPC-gene-expressing hAK cells are their proliferative and clonogenic capacities. *SIX2* overexpression significantly enhanced the proliferative capacity of adult kidney cells, where the SIX2-hKEpCs demonstrated a 2.39-fold higher growth rate than the control cells (*p* < 0.05) (Figure 1D). Additionally, the SIX2-hKEpCs exhibited significantly improved clonogenic capacity, with a 2.07-fold increase in single-cell-derived colony formation compared to the control cells (*p* < 0.05) (Figure 1E). This enhanced self-renewal capacity is consistent with the established role of *SIX2* in maintaining nephron progenitor proliferation during kidney development. In contrast, the OSR1-hKEpCs did not demonstrate a significant difference in the overall proliferation rate compared to the control cells (0.94-fold vs. control) (Figure 1D). Cells coexpressing *OSR1* and *SIX2* (OSR1+SIX2-hKEpCs) demonstrated enhanced proliferation (3.15-fold, *p* < 0.05) even beyond that of the SIX2-hKEpCs (Figure 1D), suggesting a synergistic effect between the two factors on proliferation. The clonogenic capacity of these cells was also comparable to that of SIX2-hKEpCs, with a slight additional improvement that is possibly attributable to the *OSR1*-induced stemness properties. These findings indicate the distinct functional roles of *SIX2* and *OSR1* in regulating adult kidney cell behaviors, where *SIX2* primarily enhances the proliferation and colony formation capacities while *OSR1* confers novel clonogenic potential at ultralow densities without significantly affecting the overall proliferation rate. The combination of both factors appears to have a synergistic effect, particularly on proliferation.

### *In vivo* tubulogenic potential

**FIGURE 2:**
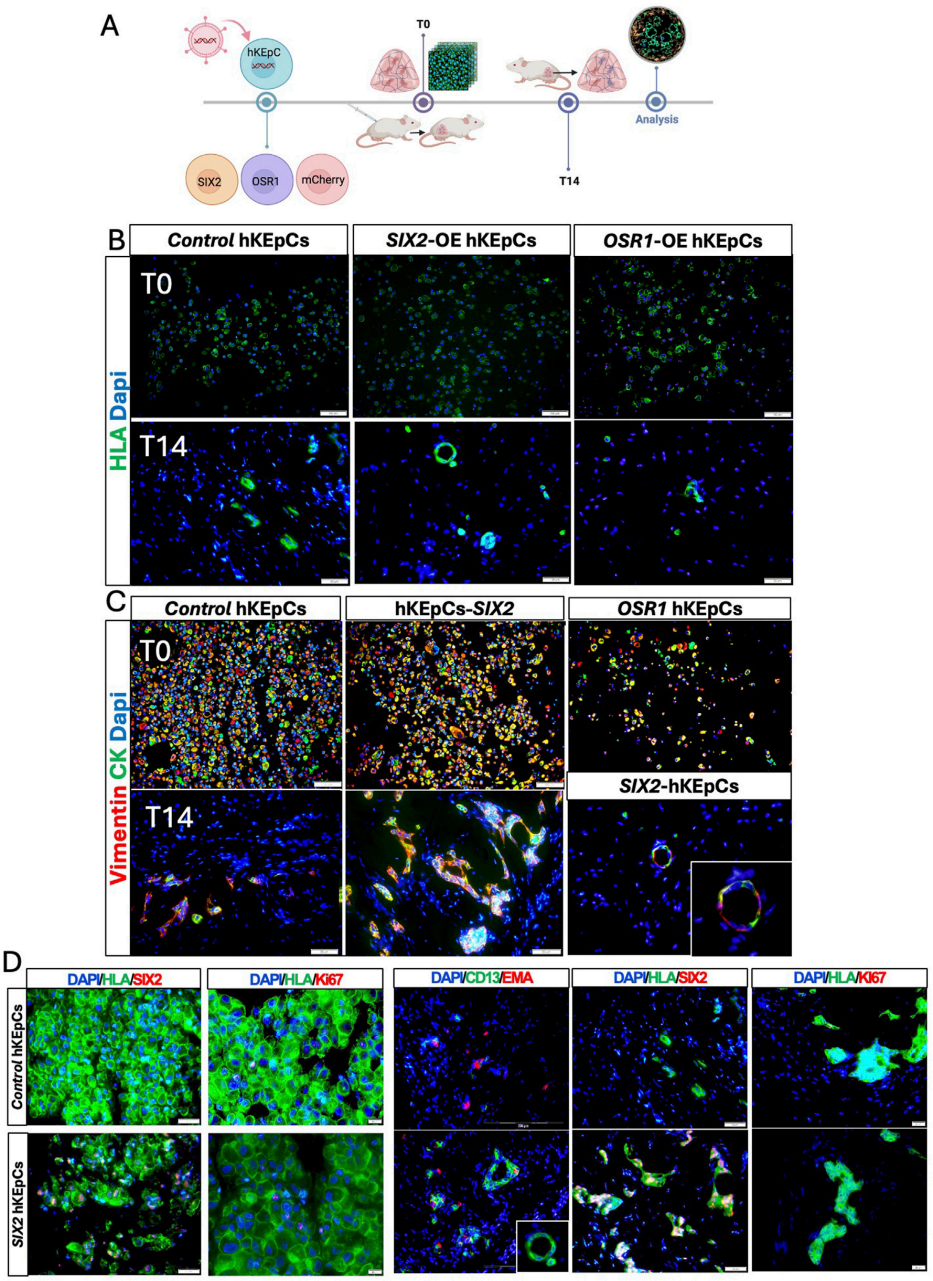
*In vivo* tubulogenic potential of the engineered hKEpCs. **(A)** Schematic representation of the *in vivo* transplantation experimental design. The engineered hKEpCs were injected subcutaneously into NOD-SCID mice and analyzed immediately (T0) as well as after 14 days (T14). **(B)** Immunofluorescence analysis using human leukocyte antigen (HLA, green) to identify the transplanted human cells at T0 (upper panels) and T14 (lower panels). At T14, the SIX2-hKEpCs formed distinct tubular structures with clear luminal organization, while the control hKEpCs formed limited disorganized cell clusters and the OSR1-hKEpCs remained as scattered cells. **(C)** Immunofluorescence staining for vimentin (red) and cytokeratin (CK, green) at T0 (upper panels) and T14 (lower panels). At T14, the SIX2-hKEpCs developed well-defined tubular structures maintaining vimentin/cytokeratin coexpression, with a high-magnification inset showing clear tubular lumen formation. **(D)** Immunofluorescence characterizations of the control hKEpCs and SIX2-hKEpCs. Left panels (T0): expressions of *SIX2* and *Ki67* in cells at the time of injection. Right panels (T14): After 14 days *in vivo*, the SIX2-hKEpCs formed CD13^+^EMA^+^ tubular structures that maintained *SIX2* expression but showed decreased proliferation, as indicated by the Ki67 staining. Representative images of results from transplantation of n = 3 primary cell types are shown, where each batch of primary cells (i.e., naïve, SIX2-overexpressing, and OSR1-overexpressing cells) was transplanted into 4–6 mice.

The tubulogenic capacities of the engineered kidney cells were assessed through subcutaneous injection in immunodeficient mice. To assess the functional implications of renal progenitor gene overexpression, we evaluated the capacities of the engineered cell lines to form tubule-like structures *in vivo*. When injected subcutaneously into immunodeficient NOD-SCID mice, all three cell populations (control hKEpCs, SIX2-hKEpCs, and OSR1-hKEpCs) from the three different hAK lines initially exhibited comparable engraftment at T0, as demonstrated by HLA staining (Figure 2B, upper panels). After 14 d (T14), striking differences were observed in the organization, with the SIX2-hKEpCs assembling into distinct tubular structures with clear luminal formation, while the control hKEpCs formed limited disorganized cell clusters and OSR1-hKEpCs primarily remained as scattered cells with minimal aggregation (Figure 2B, lower panels).

All three cell types initially expressed both vimentin and cytokeratin at T0, indicating their epithelial–mesenchymal characteristics (Figure 2C, upper panels). By T14, the SIX2-hKEpCs developed well-defined tubular structures that maintained vimentin/cytokeratin coexpression, with a representative high-magnification image showing a clear tubular lumen (Figure 2C, lower panels). After 14 d, the control hKEpCs formed mainly EMA-positive aggregates with limited tubular organization, whereas the SIX2-hKEpCs developed tubular structures with distinct luminal architecture comprising CD13^+^, EMA^+^, or occasionally CD13^+^EMA^+^ cells, with the latter possibly representing a transitional cell state (Figure 2D). The tubular structures formed by the SIX2-hKEpCs continued to express *SIX2* but showed decreased proliferation compared to T0, as indicated by Ki67 staining (Figure 2D; Supplementary Figure S2). These findings establish *SIX2* as a key driver of tubulogenic potential in adult kidney cells and promoter for formation of organized tubular structures *in vivo*, whereas *OSR1* expression alone was insufficient to confer robust tubulogenic capacity.

### Transcriptional reprogramming and pathway modulation

**FIGURE 3:**
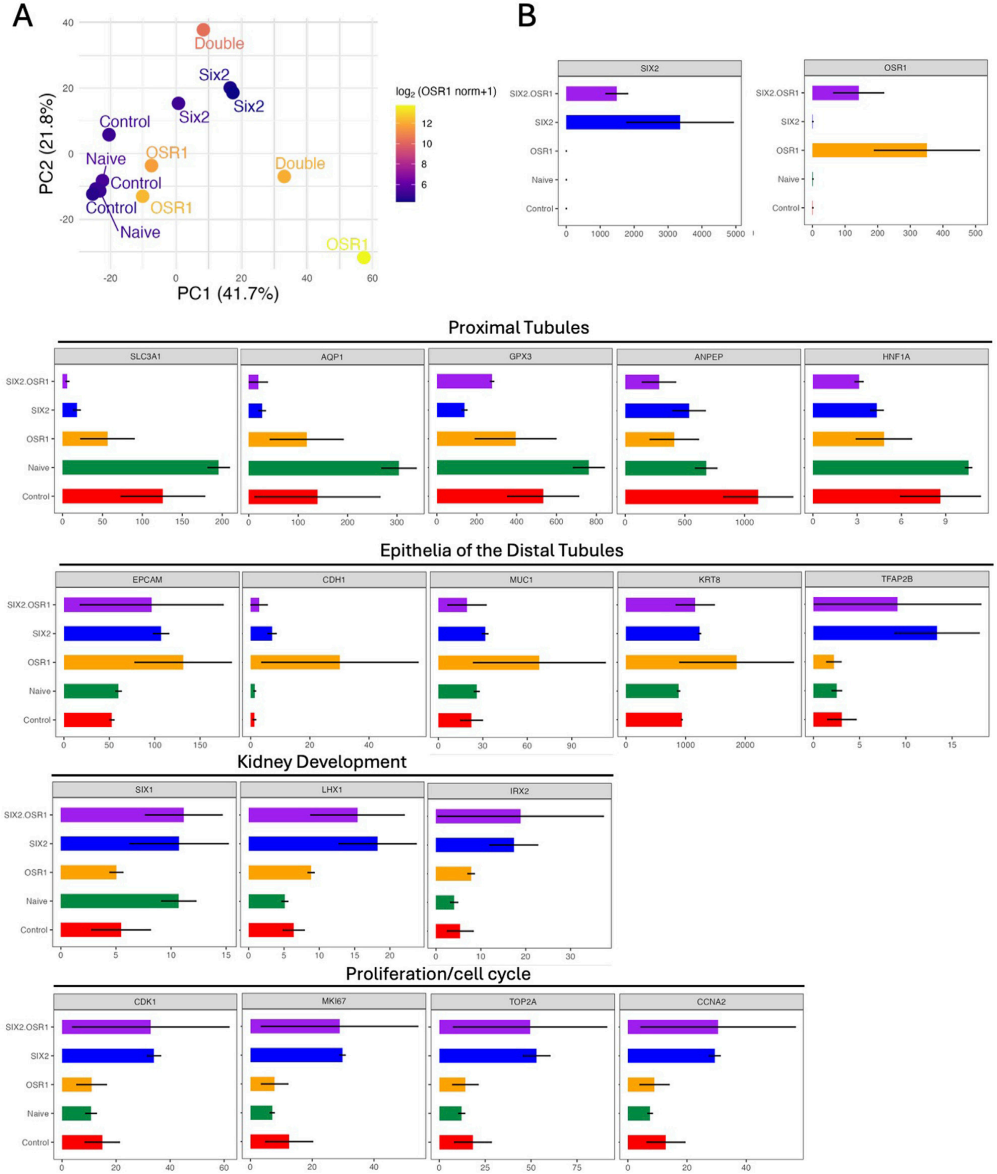
Transcriptional reprogramming and gene expression analysis. **(A)** Principal component analysis (PCA) of the RNA sequencing data shows distinct transcriptional profiles of the engineered cell lines. Each point represents a biological sample, and clear separation is noted among the naïve control cells (green), SIX2-hKEpCs (blue), OSR1-hKEpCs (orange), and OSR1+SIX2-hKEpCs (red). The first two principal components (PC1: 41.7%, PC2: 21.8%) capture the majority of transcriptional variance, demonstrating that each cell type occupies a unique position in the transcriptional landscape. **(B)** Comprehensive gene expression analysis (in transcripts per million (TPM) values) across the functional categories. The upper panels show overexpression validation, with *SIX2* and *OSR1* demonstrating the expected high expression levels in their respective cell lines. The lower panels display expression patterns across four key functional gene categories: proximal tubules showing coordinated downregulation of the mature proximal tubule markers (*SLC3A1*, *AQP1*, *GPX3*, *ANPEP*, and *HNF1A*) in both *SIX2-* and *OSR1*-expressing cells; epithelia of the distal tubules demonstrating upregulation of the distal nephron segment markers (*EPCAM*, *CDH1*, *MUC1*, *KRT8*, and *TFAP2B*), particularly in the *SIX2*-expressing cells; kidney development showing enhanced expressions of the developmental regulators (*SIX1*, *LHX1*, and *IRX2*) in the transduced cell lines; proliferation/cell-cycle mechanism revealing significant upregulation of the cell-cycle genes (*CDK1*, *MKI67*, *TOP2A*, and *CCNA2*) in the *SIX2*-expressing cells, consistent with their enhanced proliferative capacities. Color coding: SIX2+OSR1 (purple), SIX2 (blue), OSR1 (orange), Naïve (green), and Control (red).

RNA sequencing analysis revealed distinct transcriptional signatures among the cell lines overexpressing *SIX2*, *OSR1*, or both. PCA demonstrated clear separation between the cell types, with the SIX2-hKEpCs and naïve control cells forming well-defined clusters (Figure 3A). Notably, one *OSR1*-overexpressing sample clustered distantly from the other samples, potentially reflecting the heterogeneity in *OSR1*-induced cellular responses or extreme overexpression levels that may have affected transcriptional stability. Analysis of the gene expression patterns revealed coordinated changes in nephron-segment-specific markers across the overexpressing cell lines (Figure 3B). Both SIX2-hKEpCs and OSR1-hKEpCs demonstrated significant downregulation of the proximal tubule markers, including *SLC3A1*, *AQP1*, *GPX3*, *ANPEP*, and *HNF1A*, suggesting a shift away from mature proximal tubule identity. Concurrently, these cells showed upregulation of epithelialization markers correlated with distal nephron segments, such as *EPCAM*, *CDH1*, *MUC1*, *KRT8*, and *TFAP2B*, indicating reprogramming toward a more distal-tubule-like state. The SIX2-hKEpCs alone or in combination with OSR1 (SIX2+OSR1-hKEpCs) exhibited pronounced overexpression of proliferation and cell-cycle genes, including *CDK1*, *MKI67*, *TOP2A*, and *CCNA2*, which are directly correlated with the enhanced clonogenic and tubulogenic capacities observed in our functional analyses.

**FIGURE 4:**
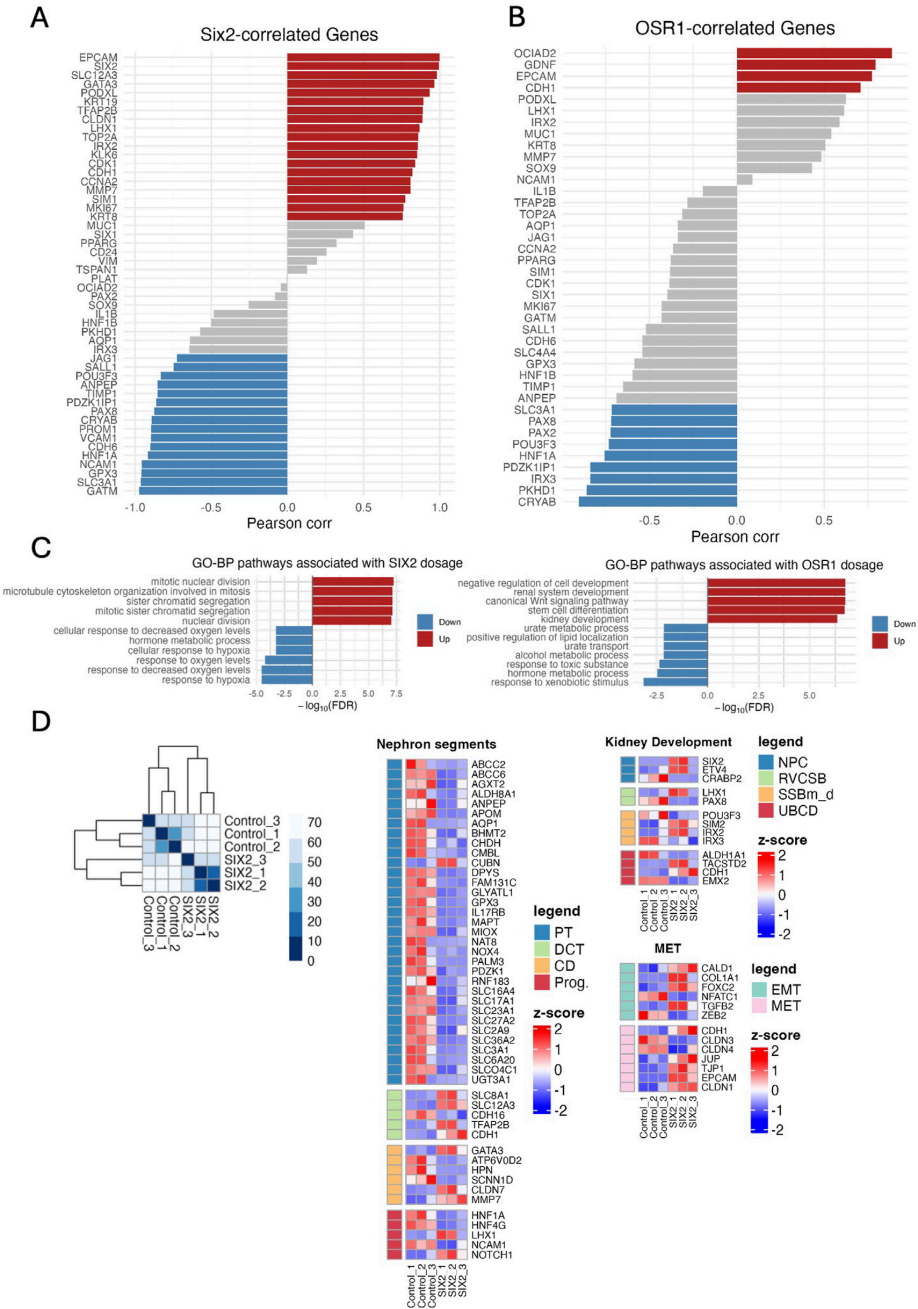
Gene correlation networks and pathway analysis. **(A)** Pearson correlation network between *SIX2* expression (log_2_ [normalized counts +1]) and a panel of nephron lineage and proliferation genes across the control and *SIX2*-overexpressing hKEpCs (n = 3 each). Strong positive correlations (r > 0.7, red) are observed for the epithelial markers (*EPCAM*, *GATA3*, *FOXD1*, and *TFAP2B*) and proliferation genes (*CDK1* and *MKI67*), whereas negative correlations (r < −0.7, blue) are noted for the progenitor genes (*SALL1* and *POU3F3*) and proximal tubule gene (*HNF1A*). The gray bars indicate intermediate correlations (|r| ≤ 0.7). This pattern highlights the roles of *SIX2* in epithelialization and proliferation as well as repression of the progenitor/proximal programs. **(B)** *OSR1* correlation network showing the Pearson correlation between *OSR1* expression (log_2_ [normalized counts +1]) and the same gene panel. Positive correlations (r > 0.7, red) are observed for the developmental/epithelial factors (*OCIAD2*, *GDNF*, and *EPCAM*), whereas negative correlations (r < −0.7, blue) are observed to be enriched for the progenitor genes (*PAX2* and *PAX8*) and proximal tubule markers (*HNF1A* and *SLC3A1*), indicating the function of OSR1 in developmental patterning. The gray bars show intermediate correlations of |r| ≤ 0.7. **(C)** Bar plots displaying GO-BP terms significantly enriched among the genes whose expressions are associated with *SIX2* (left) or *OSR1* (right) dosage, as determined by linear modeling (limma slope, false discovery rate (FDR) < –0.05, |log_2_FC| > 0.1). For *SIX2*, the enriched pathways include mitotic nuclear division, microtubule cytoskeleton organization, and cell-cycle regulation (red bars: upregulated with increasing *SIX2*), while the downregulated processes (blue bars) include cellular responses to hypoxia and hormone metabolic processes. For *OSR1*, the upregulated pathways include epithelial tube morphogenesis, renal system development, and canonical *Wnt* signaling, while the downregulated processes include cellular responses to hypoxia and amino acid metabolism. The x-axis indicates signed log_10_(FDR) and represents the statistical significance of pathway enrichment. **(D)** Left: Heatmap of the Euclidean distances between samples grouped by hierarchical clustering demonstrates separation of the control and *SIX2*-overexpressing hKEpC samples. Right: Heatmaps display z-scored and normalized expressions of the differentially expressed genes with a color scale from blue (low expression) to red (high expression). Nephron segments: Clear downregulation of the proximal tubule markers (PT - blue region) and upregulation of distal tubule markers (DCT, CD - red regions). Kidney development: Enhanced expressions of the developmental genes, including *SIX2*, *LHX1*, and other nephron patterning factors. Mesenchymal-to-epithelial transition (MET): Coordinated expression changes supporting epithelialization. The legend indicates different nephron segment identities: nephron progenitor cells (NPCs), RVCSB, SSBm_d, UBCD, proximal tubule (PT), distal convoluted tubule (DCT), collecting duct (CD), progenitor (Prog.), epithelial-to-mesenchymal transition (EMT), MET.

Gene correlation analysis revealed distinct transcriptional networks associated with *SIX2* and *OSR1* overexpressions (Figures 4A–C; Supplementary Figure S4; Supplementary Tables S1–S4). *SIX2* expression showed strong positive correlations with the epithelial markers and developmental regulators, most notably *EPCAM* (highest correlation), followed by *SLC12A3*, *GATA3*, *FOXD1*, *TFAP2B*, *CLDN1*, *LHX1*, *IRX2*, *ALK6*, *CDK1*, *MMP7*, *GPC3*, and *WNT11* (Figure 4A); these positively correlated genes suggest that *SIX2* overexpression drives epithelialization with enhanced proliferative capacity. Conversely, *SIX2* showed negative correlations with some progenitor markers like *SALL1* and *POU3F3*, proximal tubule markers like *ANPEP* and *GPX3*, and mesenchymal markers like *VCAN* and *CDH6*; this pattern indicates that *SIX2* overexpression suppresses specific progenitor programs and proximal tubule differentiation while promoting distal epithelial identity. The *OSR1*-correlated genes (Figure 4B) demonstrated positive associations with developmental factors like *OCIAD2*, *GDNF*, and *EPCAM*, while showing negative correlations with progenitor markers like *PAX2* and *PAX8* as well as the proximal tubule genes *HNF1A* and *SLC3A1*, indicating distinct regulatory networks activated by each transcription factor.

Pathway enrichment analysis revealed differential activation of the BPs between the transcription factors (Figure 4C). *SIX2* overexpression was enriched for pathways related to mitotic nuclear division, microtubule cytoskeleton organization, and cell-cycle regulation while being downregulated for cellular responses to decreased oxygen levels and hormone metabolic processes. In contrast, *OSR1* overexpression was upregulated for pathways involved in epithelial tube morphogenesis, renal system development, and canonical *Wnt* signaling while being downregulated for metabolic processes like cellular responses to hypoxia and amino acid metabolism. Given the superior functional performance of the SIX2-hKEpCs in both *in vitro* proliferation assays and *in vivo* tubulogenic studies, we conducted focused transcriptional analysis of these cells (Figure 4D). Hierarchical clustering analysis revealed distinct expression patterns across key functional categories, including kidney development, nephron segments, EMTs, and metabolic processes. Detailed analyses of the nephron segment markers confirmed that the SIX2-hKEpCs specifically downregulated proximal tubule genes (*SLC3A1*, *AQP1*, *GPX3*, *ANPEP*, and *HNF1A*) while maintaining or upregulating certain distal tubule markers (*EPCAM*, *CDH1*, *MUC1*, *KRT8*, and *TFAP2B*). The kidney developmental genes, including *SIX2*, *SIX1*, *LHX1*, and *IRX2*, showed enhanced expressions while the proliferation/cell-cycle markers like *CDK1*, *MKI67*, *TOP2A*, and *CCNA2* were significantly upregulated, consistent with the enhanced proliferative capacity observed functionally. In summary, the addition of *SIX2* or *OSR1* to adult kidney cells can reprogram their gene expression profile away from a mature proximal tubule phenotype toward an epithelialized state with the features of distal nephron segments. *SIX2* alone or with *OSR1* can concomitantly enhance the proliferative genes to support improved self-renewal and tubulogenic functions.

### Malignant transformation associated with *OSR1* overexpression

**FIGURE 5:**
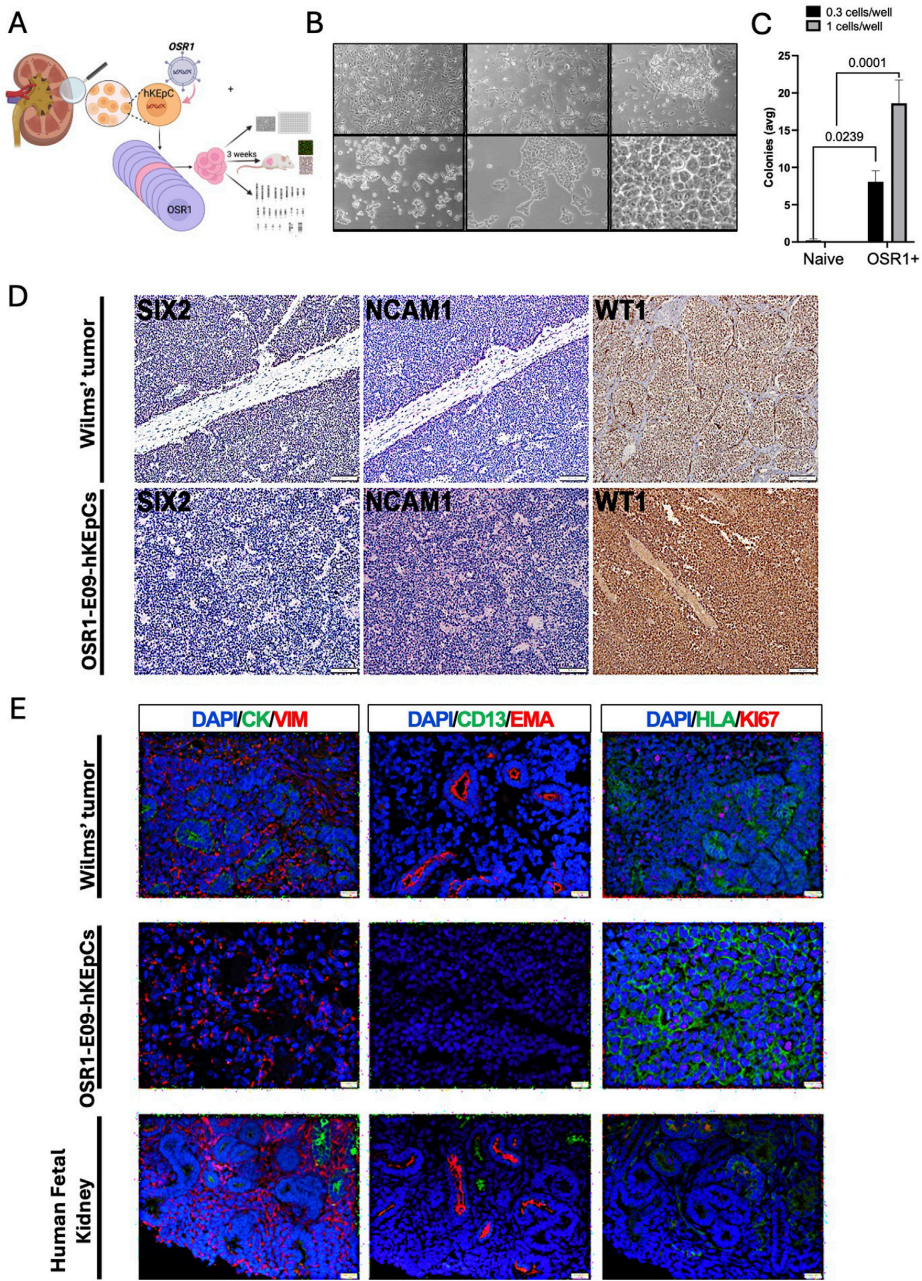
Malignant transformation associated with *OSR1* overexpression. **(A)** Schematic representation of the experimental approach to characterize the unique OSR1-hKEpCs clone (E09) that demonstrated tumorigenic capacity. The OSR1-hKEpCs were first evaluated for their clonogenic potential through ultralow-density seeding experiments. The OSR1-E09 clone was then expanded, characterized *in vitro*, and injected subcutaneously into NOD-SCID mice to assess the *in vivo* behaviors. The resulting tumor masses were harvested after approximately 3 weeks and analyzed through histological and immunofluorescence methods to compare their features with those of Wilms’ tumor and the human fetal kidney. **(B)** Representative phase-contrast microscopy images of the OSR1-E09-hKEpCs forming colonies at different densities, demonstrating their unique morphological characteristics with dense, hyperchromatic cells forming distinct colony-like structures. **(C)** Quantification of colony formation by the naïve-hKEpCs and OSR1-hKEpCs when seeded at ultralow densities (0.3 and 1 cell/well). The OSR1-hKEpCs uniquely acquire the ability to form colonies at these densities, which is a capacity absent in the control cells. The results are presented as mean ± SEM of n = 3 replicates, and the *p*-values are indicated in the image. **(D)** Immunohistochemical analyses of Wilms’ tumor (upper panels) and OSR1-E09-hKEpC-derived mass (lower panels) showing expressions of the renal developmental markers *SIX2*, *NCAM1*, and *WT1*. Both the Wilms’ tumor and OSR1-E09-hKEpC-derived mass demonstrate similar expression patterns of these markers. **(E)** Comparative immunofluorescence analysis of Wilms’ tumor (top row), OSR1-E09-hKEpC-derived mass (middle row), and human fetal kidney (bottom row). Left column: vimentin (VIM, red) and cytokeratin (CYTO, green) staining shows the mesenchymal phenotype of the *OSR1*-derived mass with vimentin^+^/cytokeratin^–^ expression similar to that of Wilms’ tumor. Middle column: EMA (red) and CD13 (green) staining reveals the absence of mature renal epithelial markers in the *OSR1*-derived mass. Right column: Ki67 (red) and HLA (green) staining demonstrates high proliferative activity in the *OSR1*-derived mass comparable to that of Wilms’ tumor. The OSR1-hKEpCs show positive staining for the proliferation marker Ki67, similar to the blastema of Wilms’ tumor.

A strikingly unexpected finding of this study was that *OSR1* overexpression led to malignant transformation in approximately one in a hundred OSR1-hKEpC-transduced clones. This discovery was based on the identification of a unique clone, OSR1-E09-hKEpCs, which exhibited distinct morphological characteristics that prompted further investigation (Figures 5A,B). Unlike the other OSR1-hKEpCs clones, this particular clone displayed exceptional growth and markedly different morphological features, prompting us to conduct a more detailed characterization. Karyotype analysis of the transformed cells revealed multiple chromosomal abnormalities affecting both the structure and numbers across multiple chromosomes, confirming cellular transformation (Supplementary Figure S4H). Importantly, sequencing analysis of the viral insertion site showed no disruption of any known oncogenes or tumor suppressor genes, suggesting that the transformation was likely due to the *OSR1* overexpression itself rather than insertional mutagenesis (Supplementary Figure S4I). When injected subcutaneously into NOD-SCID mice, the OSR1-E09-hKEpCs clone formed tumors within approximately 3 weeks. Histological examination of these tumors showed a structure characterized by dense sheets of poorly differentiated cells with a hyperchromatic morphology, high nucleus-to-cytoplasm ratios, and evident mitotic activity (Supplementary Figure S4J) that closely resembled the blastemal component of WT.

Immunohistochemical characterization revealed that these tumors exhibited a strikingly similar molecular profile to WT. Both the OSR1-E09-hKEpC-derived masses and WT samples expressed key renal developmental markers, including *SIX2*, *NCAM1*, and *WT1* (Figure 5D). A comparison between WT, OSR1-E09-hKEpC-derived masses, and human fetal kidney tissue revealed further similarities in the phenotypic profiles (Figure 5E). The tumors were exclusively mesenchymal, expressing vimentin but not cytokeratin (Figure 5E, left column), and lacked mature renal epithelial markers like *EMA* and *CD13* (Figure 5E, middle column). Additionally, the tumors showed robust positive staining for the proliferation marker Ki67 (Figure 5E, right column), comparable to the proliferative activity observed in the blastemal component of WT. Importantly, this malignant transformation phenomenon was not observed in either the SIX2-hKEpCs or OSR1+SIX2-hKEpCs, suggesting that it is specific to *OSR1* overexpression and may be mitigated by concomitant *SIX2* expression. The relatively low transformation frequency (approximately one in 100 *OSR1*-transduced clones) suggests that additional factors may influence susceptibility to malignant transformation, possibly including stochastic genetic or epigenetic alterations that cooperate with *OSR1* overexpression to drive tumorigenesis.

## Discussion

The present study demonstrates that ectopic expression of the key nephron development transcription factors OSR1 and SIX2 in hAK cells induces distinct reprogramming processes with differential functional outcomes. Our comprehensive analysis reveals that *SIX2* enhances proliferative capacity while maintaining the epithelial traits and conferring tubulogenic potential, whereas *OSR1* induces morphological changes and developmental reprogramming but may pose oncogenic risks. The effects of *OSR1* and *SIX2* on hAK cells reflect their different developmental roles. *OSR1* overexpression induced significant morphological changes toward a cuboidal epithelial-like phenotype, with cells developing dense hyperchromatic morphology and forming well-defined colonies of small cuboidal cells. Despite these morphological alterations, the OSR1-hKEpCs did not demonstrate a significant difference in the overall proliferation rate compared to the control cells (0.94-fold vs. control). In contrast, *SIX2* overexpression maintained a typical epithelial morphology with minimal morphological changes while significantly enhancing the proliferative capacity (2.39-fold higher growth rate, *p* = 0.045) and clonogenic efficiency (2.07-fold increase in single-cell-derived colony formation, *p* = 0.01), consistent with its established role in maintaining nephron progenitor self-renewal during development (Kobayashi et al., 2008). Cells coexpressing *OSR1* and *SIX2* demonstrated enhanced proliferation (3.15-fold higher rate, *p* = 0.03) beyond that of either factor alone, suggesting synergistic effects between the two factors.

Although our study demonstrates robust reprogramming effects following *OSR1* and *SIX2* overexpression, we acknowledge that non-tumoral adult kidney tissues can harbor rare populations of adult renal progenitor cells (ARPCs) (Romagnani et al., 2013; Bussolati et al., 2005; Sallustio et al., 2010). Our isolation protocol did not involve enrichment or depletion of these progenitor populations, and our culture conditions included growth factors that could theoretically favor progenitor cell survival. However, the magnitude of transcriptional remodeling and functional enhancement observed in our experiments, particularly in the *SIX2*-expressing cells, supports reprogramming of a broader epithelial population than that expected from the expansion of rare ARPCs alone. While we cannot exclude the presence of a small fraction of ARPCs in the starting population, the overall magnitude and reproducibility of the effects are indicative of an induced shift in cell identity rather than selection or expansion of preexisting progenitors.

The tubulogenic capacity of the *SIX2*-expressing cells *in vivo* represents a functionally significant finding that provides initial insights into potential cellular reprogramming mechanisms. The formation of organized tubular structures expressing proximal (*CD13*), distal (*EMA*), or both tubular markers along with epithelial (E-cadherin and cytokeratin) and mesenchymal (vimentin) markers demonstrates that *SIX2* overexpression can confer specific differentiation capacity to adult kidney cells while maintaining the cellular plasticity characteristic of developing nephrons. This marker expression pattern suggests that *SIX2*-reprogrammed cells retain the ability to express markers from different nephron segments simultaneously in a manner reminiscent of the transitional states during normal nephrogenesis. The coexpression of both epithelial and mesenchymal markers reflects the mesenchymal-to-epithelial transition that occurs during kidney development, where the NPCs retain vimentin expression while acquiring epithelial characteristics. This hybrid phenotype may provide the structural flexibility required during tubule formation and organization. To evaluate the tubulogenic potential *in vivo*, we employed subcutaneous Matrigel transplantation as a widely accepted model (Machiguchi and Nakamura, 2019; Pleniceanu et al., 2020). Although this approach effectively demonstrates the structural organizational capacity conferred by *SIX2* overexpression, the ectopic environment lacks the features of the native renal microenvironment, including stromal interactions, vascular supply, and signaling gradients. Additionally, this model focuses on structural tubule formation rather than functional integration (Harari-Steinberg et al., 2020). Nevertheless, our subcutaneous model provides valuable proof-of-concept data demonstrating enhanced tubulogenic capacity following *SIX2* overexpression.

Our comprehensive RNA sequencing analyses revealed that *OSR1* and *SIX2* activated different transcriptional programs in hAK cells. The transcriptional reprogramming induced by each of these factors involved a coordinated shift away from mature proximal tubule identity, as evidenced by the significant downregulation of proximal tubule markers like *SLC3A1*, *AQP1*, *GPX3*, *ANPEP*, and *HNF1A*. Concurrently, both cell types showed upregulation of the epithelialization markers associated with distal nephron segments, such as *EPCAM*, *CDH1*, *MUC1*, *KRT8*, and *TFAP2B*, suggesting that both *OSR1* and *SIX2* drive reprogramming toward a more primitive or distal-tubule-like state. This interpretation is further supported by our immunophenotyping analysis of the epithelial markers (cytokeratin, E-cadherin) that confirms the maintenance of epithelial characteristics during reprogramming. Future single-cell profiling approaches could provide additional insights into the cellular heterogeneity and transitional states during this reprogramming process.

Gene correlation analysis revealed that *SIX2* expression establishes a robust transcriptional network centered on epithelialization and proliferation, with the strongest positive correlation observed with *EPCAM*, followed by developmental regulators like *SLC12A3*, *GATA3*, *FOXD1*, and *TFAP2B* as well as proliferation-associated genes like *CDK1*, *MKI67*, *TOP2A*, and *CCNA2*. This transcriptional signature is directly correlated with the enhanced clonogenic and tubulogenic capacities observed functionally. Pathway enrichment analysis further supported this explanation, showing that *SIX2* overexpression specifically enriched the pathways related to mitotic nuclear division, microtubule cytoskeleton organization, and cell-cycle regulation while downregulating metabolic pathways like cellular responses to decreased oxygen levels and hormone metabolism. In contrast, the *OSR1*-correlated genes demonstrated positive associations with developmental morphogenetic factors like *GDNF* and epithelial tube morphogenesis regulators while showing negative correlations with both progenitor markers (*PAX2* and *PAX8*) and proximal tubule genes (*HNF1A* and *SLC3A1*). Accordingly, pathway enrichment analysis revealed that *OSR1* overexpression was indeed associated with upregulation of epithelial tube morphogenesis, renal system development, and the canonical *Wnt* signaling pathways as well as downregulation of metabolic processes. The coordinated downregulation of oxidative phosphorylation and fatty acid metabolism pathways in both cell types indicates a fundamental shift away from mature kidney cell metabolism that could facilitate the energetic demands of enhanced proliferation while removing the metabolic constraints that maintain differentiated cellular states.

The unexpected finding that *OSR1* overexpression led to malignant transformation in approximately one in 100 transduced clones offers insight into the potential mechanisms underlying WT development. The histological and molecular features of these tumors, including expression of renal developmental markers (*SIX2*, *NCAM1*, and *WT1*), expression of the mesenchymal phenotype (vimentin^+^/cytokeratin^–^), and lack of mature epithelial markers (EMA^–^/CD13^–^), bear resemblance to those of WT (Pode-Shakked and Dekel, 2011; Hohenstein et al., 2015). The transcriptional networks activated by *OSR1*, particularly those involving epithelial tube morphogenesis and canonical *Wnt* signaling, are known to be dysregulated in various cancers, suggesting that aberrant *OSR1* expression may contribute to tumorigenesis by activating developmental programs inappropriate for adult cellular contexts. The high proliferative activity indicated by Ki67 staining further supports this and is aligned with the hypothesis that WT arises from aberrant developmental processes in the renal progenitor cells (Pode-Shakked and Dekel, 2011; Petrosyan et al., 2023). While the malignant transformation observed with *OSR1* overexpression was limited to a single clone (∼1% frequency), several lines of evidence suggest that this occurrence may be more than a purely stochastic event. First, comprehensive lentiviral insertion site analysis revealed that viral integration occurred in an intronic region of *HECTD4* rather than disrupting known oncogenes or tumor suppressor genes, arguing against insertional mutagenesis as the primary cause. Second, the transformed clone specifically developed WT-like characteristics, including expression of renal developmental markers (*SIX2*, *NCAM1*, and *WT1*) and a blastemal morphology. If this transformation were merely stochastic and unrelated to *OSR1* function, we would expect a random tumor type, most likely renal cell carcinoma given the adult kidney origin of our engineered cells. The specific development of a WT-like phenotype suggests a mechanistic connection to the role of *OSR1* in renal development and its known associations with pediatric kidney cancer pathways. Finally, our transcriptomic analysis demonstrates that *OSR1* overexpression activates the developmental pathways known to be dysregulated in WT, including the canonical *Wnt* signaling and epithelial tube morphogenesis networks. Nevertheless, the low frequency and single-clone nature of this observation precludes definitive conclusions about the oncogenicity of *OSR1*, which requires further validation through independent biological replicates and functional assays to establish causality (Humphreys, 2018).

The differential effects observed between *OSR1* and *SIX2* suggest distinct considerations for potential regenerative applications. *SIX2* overexpression that enhanced the proliferation and tubulogenic capacities in our system without notable malignant transformation provides proof-of-concept data that may inform future approaches to generate renal progenitor populations. The robust transcriptional networks established by *SIX2*, particularly those driving epithelialization and proliferation, provide a molecular framework for understanding its regenerative potential. Conversely, the oncogenic potential associated with *OSR1* overexpression, combined with its activation of the developmental morphogenetic programs that may be inappropriate in adult contexts, highlights important safety considerations that need to be addressed for future regenerative medicine approaches involving *OSR1* induction in adult kidney cells.

Aside from the critical findings presented herein, our study has several limitations. First, we need to consider the use of cytomegalovirus promoters for gene overexpression as these may induce higher expressions than those observed physiologically. However, benchmarking against published fetal kidney transcriptomes shows that our observed expression levels are within the fold range of native NPCs (O'Brien et al., 2016). While most cells can tolerate these expression levels well, the low *OSR1* transformation frequency (1 in 100 clones) suggests that occasional extreme expressions may trigger stress responses. Second, our exclusive use of overexpression models without complementary loss-of-function validation is another consideration as it cannot definitively establish the necessity of *SIX2* for tubulogenesis or suppression of *OSR1* for reversing the malignant characteristics. The *in vitro* nature of most experiments may not fully recapitulate the complex microenvironment of the kidney and its influence on the transcriptional programs. Additionally, our transcriptional analysis captures only the steady-state transcript levels rather than dynamic transcriptional changes during reprogramming. Although the use of immunodeficient mice is necessary for xenograft experiments, it does not account for the immune responses that might influence cell behaviors in clinical settings; further, the relatively short durations of our *in vivo* studies limit assessments of the long-term safety and functionality of the reprogrammed cells. Thus, future studies will need to address the long-term safety, reprogramming reversibility, and functional integration potential within native kidney environments to determine the translational viability.

In conclusion, our study demonstrates that *SIX2* overexpression in primary hAK cells can functionally confer enhanced self-renewal and tubulogenic capacities while transcriptionally revealing a unique combination of enhanced cell-cycle/proliferation programs and maintenance of epithelial traits. Specifically, *SIX2* shifts the cells away from mesenchymal characteristics and a proximal tubular identity (associated with dedifferentiation) toward distal tubular epithelial features, providing insights into transcription factor-induced cellular plasticity. In contrast, *OSR1* activates the broader developmental morphogenetic networks but does not confer tubulogenic potential; it also poses potential oncogenic risks that should be considered carefully. These findings provide critical insights into the molecular mechanisms underlying developmental-gene-mediated cellular reprogramming and help establish a comprehensive framework for understanding how transcription-factor-directed reprogramming can be optimized for renal regeneration, highlighting both the therapeutic potential and safety considerations for regenerative approaches targeting adult kidney cells.

## Data Availability

The RNA sequencing data generated and analyzed in this study have been deposited in the NCBI Gene Expression Omnibus (GEO) under accession number GSE309076. The submission includes gene-level count matrices, normalized expression values, and differential expression results, together with complete metadata. Raw sequencing reads are not available. All other data supporting the findings of this study are included in the article and its Supplementary Material.
