# Supplementary material for: OSR1 and SIX2 drive divergent transcriptional programs in human kidney cells: implications for regeneration and tumorigenesis
Source: Front Bioeng Biotechnol. 2025 Oct 3;13:1645499. doi: 10.3389/fbioe.2025.1645499 (PMC12531215; doi:10.3389/fbioe.2025.1645499)
Supplement: Supplementary file 3 [file DataSheet1.pdf]

Supplemental information – Table of content:

SUPPLEMENTAL FIGURES AND LEGENDS 2

FIGURE S1| *ESTABLISHMENT AND VALIDATION OF KIDNEY PROGENITOR GENE-OVEREXPRESSING HUMAN KIDNEY EPITHELIAL CELLS* 2

FIGURE S2| Tubulogenic potential of SIX2-hKEpCs 4

FIGURE S3|Gene Expression Heatmaps of SIX2-hKEpCs and OSR1-hKEpCs 5

FIGURE S4| OSR1-hKEpC-E09 Characterization and Malignant Transformation 6

SUPPLEMENTAL TABLES 7

TABLE S1: LIST OF CELL LINES ESTABLISHED BY LENTIVIRAL INTRODUCTION OF SIX2 AND OSR1 INTO HUMAN KIDNEY EPITHELIAL CELLS 7

Supplemental Figure S1

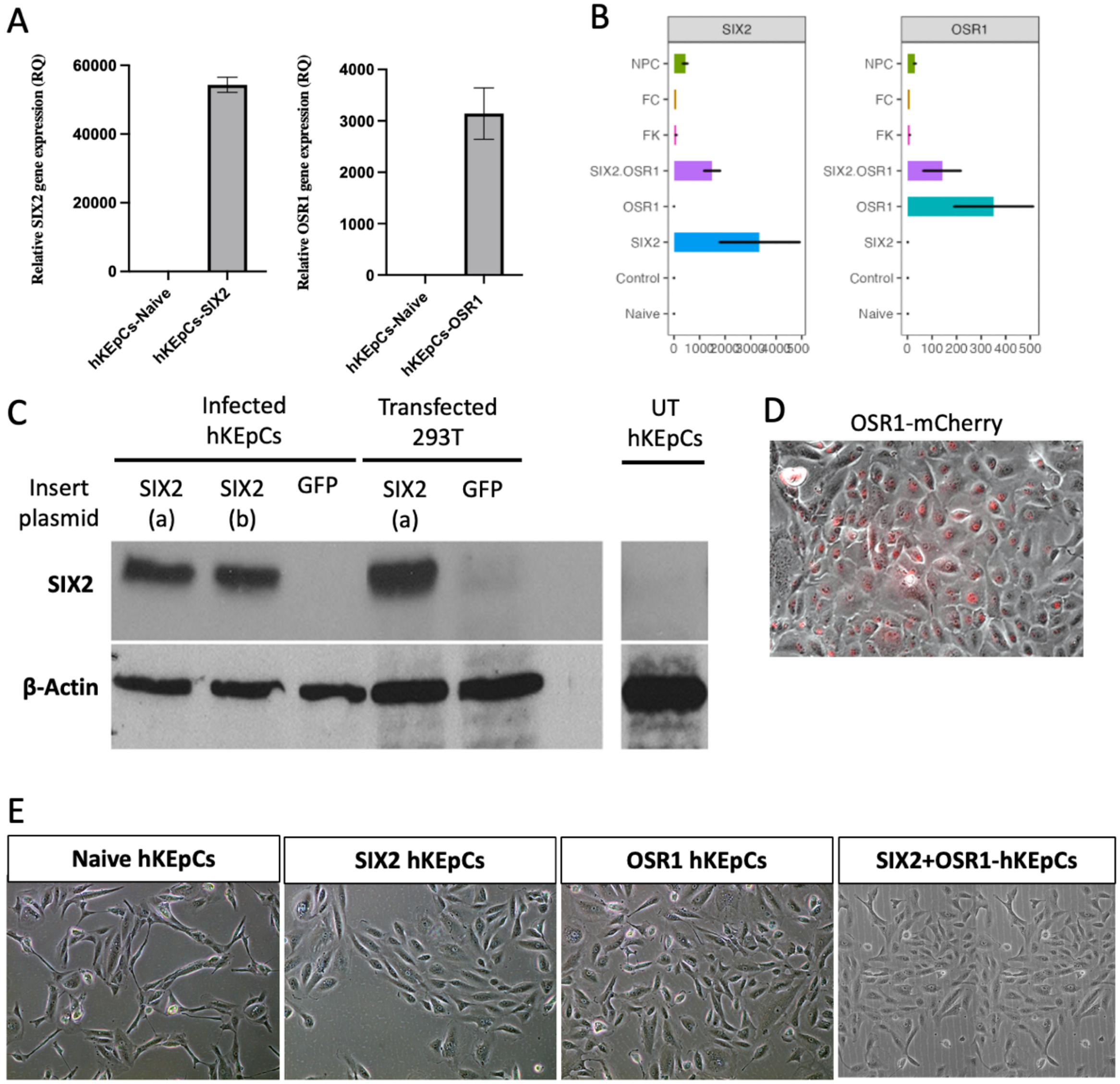

Establishment and Validation of Kidney Progenitor Gene-Overexpressing Human Kidney Epithelial Cells

A. Quantitative real-time PCR analysis confirming successful overexpression of SIX2 (left panel) and OSR1 (right panel) in transduced human kidney epithelial cells (hKEpCs) compared to control cells. SIX2 shows approximately 50,000-fold increased expression in SIX2-transduced cells, while OSR1 demonstrates approximately 3,000-fold overexpression in OSR1-transduced cells. Data presented as relative gene expression (RQ) normalized to control cells. Error bars represent standard deviation from triplicate experiments. B. Comparative analysis with human fetal kidney populations. Expression levels of SIX2 and OSR1 in our overexpressing cells are within the same fold scale as those observed in purified ITGA8^{+} nephron progenitor cells (NPCs) from human fetal kidneys[12]. C. Western blot analysis confirming SIX2 protein expression in transduced hKEpCs. SIX2 protein (~32 kDa) is detected in SIX2-transduced cells using both lentiviral constructs (SIX2-a and SIX2-b) but not in GFP-transduced control cells or untransduced (UT) hKEpCs. β-Actin serves as a loading control (~42 kDa). The analysis includes infected hKEpCs, transfected 293T cells (positive control), and untransduced hKEpCs (negative control). D. Representative fluorescent microscopy image

demonstrating OSR1-mCherry expression in OSR1-transduced hKEpCs. The mCherry fluorescence (red signal) is visible throughout the cytoplasm and nuclei of transduced cells, confirming successful expression of the OSR1-mCherry fusion protein. E. Representative phase-contrast microscopy images showing the distinct morphological characteristics of each cell line. Naïve hKEpCs display typical epithelial morphology with cobblestone-like appearance. SIX2-hKEpCs maintain epithelial characteristics with slightly more compact cellular organization. OSR1-hKEpCs show altered morphology with denser, more hyperchromatic cells forming distinct colonies. SIX2+OSR1-hKEpCs exhibit intermediate morphological features combining characteristics of both single-factor cell lines. All images captured at the same magnification for direct morphological comparison.

Supplemental Figure S2

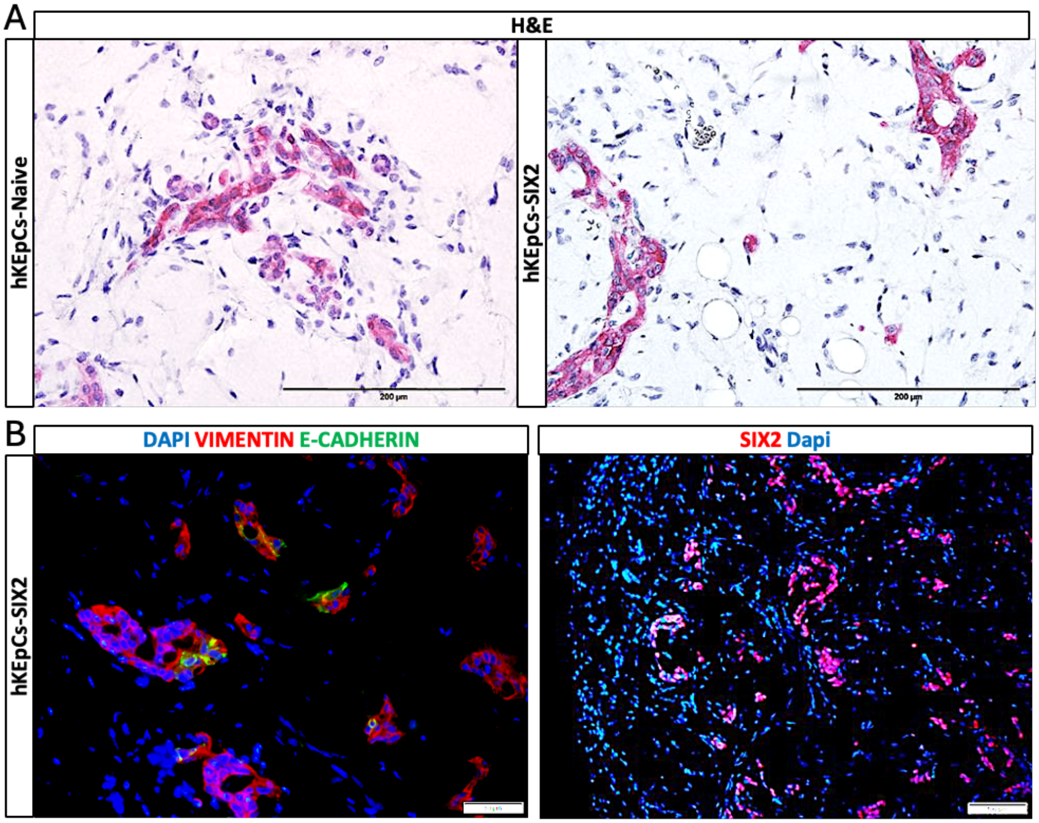

A. H&E staining of subcutaneous transplants at 14 days post-injection. Left panel shows hKEpCs-Naïve forming disorganized cellular aggregates with minimal structural organization. Right panel shows hKEpCs-SIX2 developing more organized tubular-like structures with improved cellular arrangement and morphology. Scale bars = 200 µm.

B. Immunofluorescence analysis of transplanted cells at 14 days. Left panel: Triple staining for DAPI (blue, nuclei), vimentin (red, mesenchymal marker), and E-cadherin (green, epithelial marker) in hKEpCs-SIX2 transplants showing co-expression of both epithelial and mesenchymal markers in organized structures. Right panel: DAPI (blue) and SIX2 (red) staining demonstrating continued expression of the SIX2 transcription factor in transplanted cells, confirming maintenance of the engineered phenotype in vivo. Scale bars = 50 µm.

Supplemental Figure S3

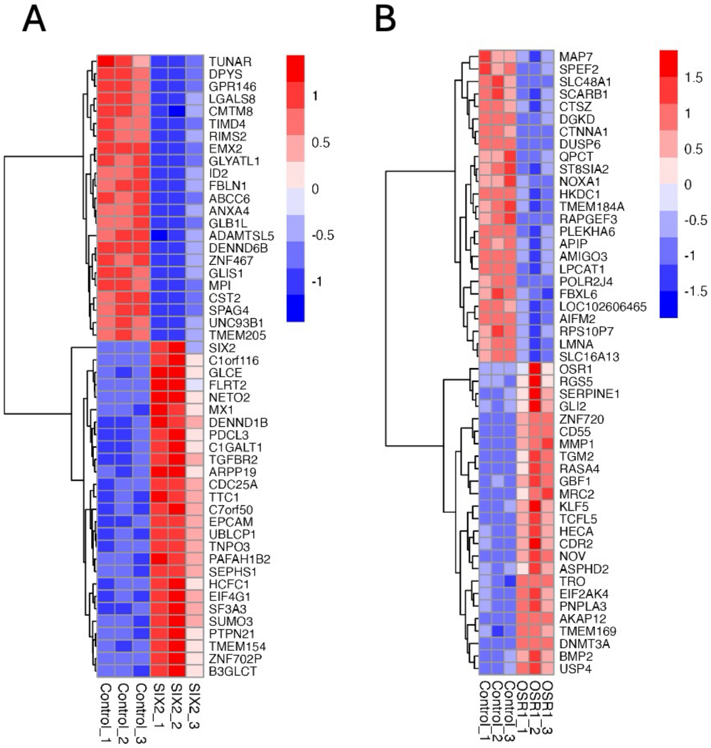

Gene Expression Heatmaps of SIX2-hKEpCs and OSR1-hKEpCs

A. Hierarchical clustering heatmap showing differential gene expression patterns in SIX2-hKEpCs compared to control cells. The heatmap displays z-score normalized expression values with red indicating upregulated genes and blue indicating downregulated genes. Genes are clustered based on expression similarity, revealing coordinated regulation of gene sets. Notable upregulated genes include developmental factors and cell cycle regulators, while downregulated genes include metabolic enzymes and mature epithelial markers. Sample replicates are shown across columns (Control_1, Control_2, Control_3, SIX2_1, SIX2_2, SIX2_3) demonstrating reproducibility across biological replicates.

B. Hierarchical clustering heatmap displaying differential gene expression in OSR1-hKEpCs versus control cells. The expression pattern shows distinct clustering from SIX2-hKEpCs, with OSR1-specific upregulation of developmental morphogenesis genes and downregulation of metabolic pathways. The heatmap reveals that OSR1 and SIX2 activate largely non-overlapping gene expression programs, supporting their distinct functional roles. Sample organization follows the same pattern as panel A, with control and OSR1-expressing cell replicates grouped by hierarchical clustering. Color scale represents z-score normalized expression levels ranging from high expression (red, +1.5) to low expression (blue, -1.5).

Supplemental Figure S4

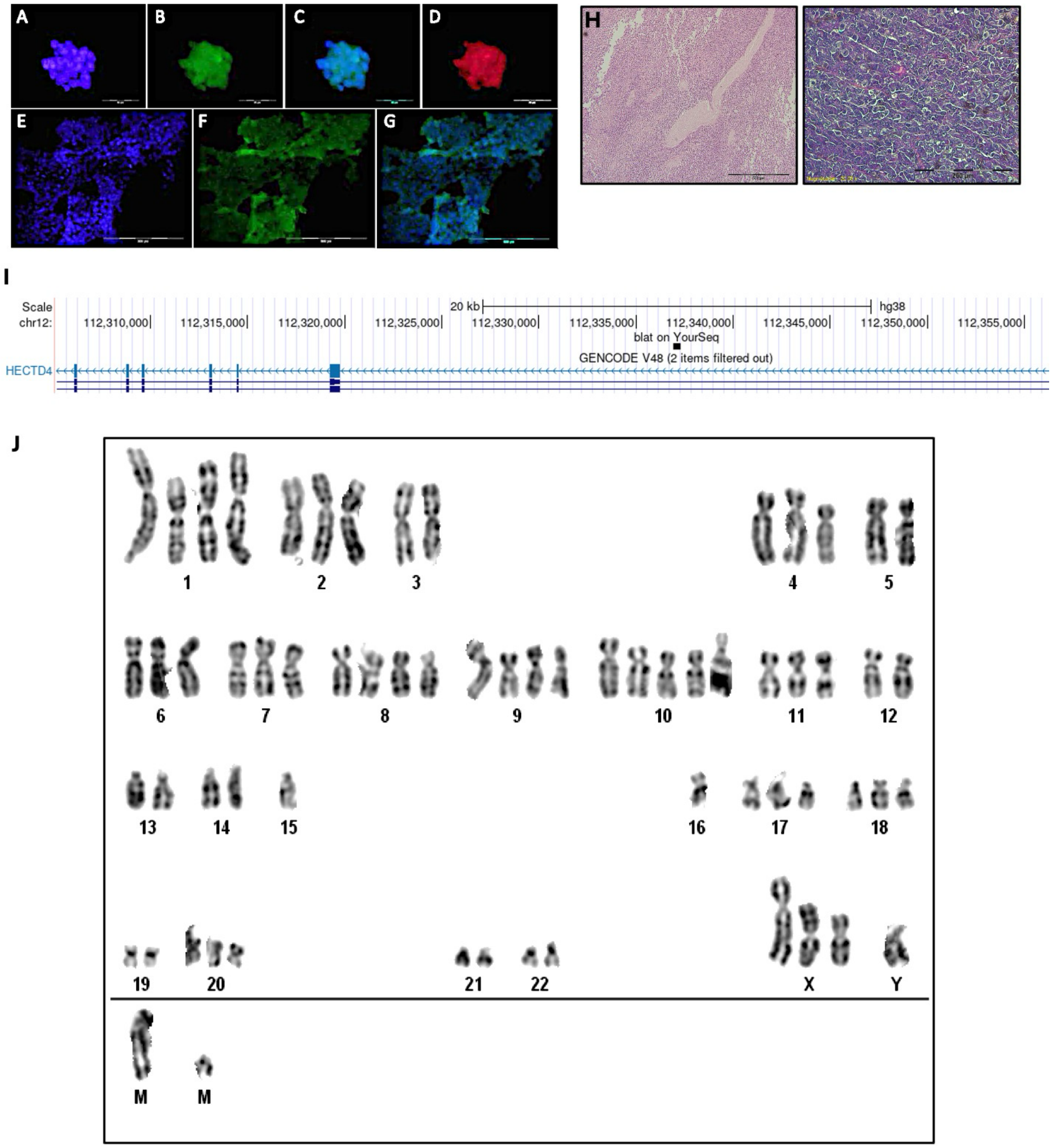

OSR1-hKEpC-E09 Characterization and Malignant Transformation

A-G. Immunofluorescence characterization of OSR1-hKEpCs demonstrating expression of key kidney developmental markers. **A-C:** WT1 staining showing nuclear localization pattern (A: DAPI nuclear stain, B: WT1 immunofluorescence, C: merged overlay). **D:** Expression of mCherry reporter confirming OSR1-mCherry fusion protein expression throughout the cell. **E-G:** SIX2 staining demonstrating nuclear localization (E: DAPI, F: SIX2 immunofluorescence, G: merged overlay), indicating activation of nephron progenitor markers in OSR1-expressing cells. Scale bars indicate appropriate magnification for subcellular detail visualization.

H. Histological analysis of tumors formed by OSR1-E09-hKEpCs in NOD-SCID mice approximately three weeks post-injection. **Left panel:** Low magnification H&E staining showing dense sheets of poorly differentiated cells with characteristics reminiscent of Wilms' tumor blastemal component. **Right panel:** High magnification view demonstrating hyperchromatic cells with high nucleus-to-cytoplasm ratios, prominent nucleoli, and evident mitotic activity, confirming the malignant nature of the transformation.
I. Lentiviral integration site analysis of OSR1-hKEpC-E09 using targeted sequencing around LTR regions. The integration site is located within an intronic region of HECTD4 (probable E3 ubiquitin-protein ligase HECTD4) on chromosome 12, indicating that the malignant transformation observed in this clone is not due to insertional mutagenesis but rather results from other genetic alterations.
J. Karyotype analysis of OSR1-hKEpC-E09 demonstrating multiple chromosomal abnormalities confirming cellular transformation. The karyogram shows various structural and numerical chromosomal aberrations across multiple chromosomes, including translocations, deletions, and aneuploid changes. These chromosomal instabilities are consistent with malignant transformation and distinguish this clone from the stable diploid karyotype expected in normal primary human kidney epithelial cells. The complex karyotype supports the "multiple hit" model of cancer development, where OSR1 overexpression cooperates with additional genetic alterations to drive tumorigenesis.

Supplemental table S1: List of cell lines established by lentiviral introduction of renal progenitor genes (SIX2 and OSR1) into human kidney epithelial cells*

**Over-Expressed Gene**	**Eukaryotic Selection**	**Fluorescent Reporter**	**Designation**
SIX2	Puromycin	None	SIX2-hKEpC
OSR1	Puromycin	mCherry	OSR1-hKEpC
mCherry	Puromycin	mCherry	Naive-hKEpC
OSR1 and SIX2	Neomycin and Puromycin	mCherry	OSR1+SIX2-hKEpC

* Each of these lines were generated from n=3 primary human adult kidney cells (hAK83, hAK86, hAK87)
